# Central activation of the fatty acid sensor GPR120 suppresses microglia reactivity and alleviates sickness- and anxiety-like behaviors

**DOI:** 10.1186/s12974-023-02978-5

**Published:** 2023-12-19

**Authors:** Shingo Nakajima, Geneviève Demers, Arturo Israel Machuca-Parra, Zahra Dashtehei Pour, Diane Bairamian, Khalil Bouyakdan, Alexandre Fisette, Anita Kabahizi, Josephine Robb, Demetra Rodaros, Cyril Laurent, Guillaume Ferreira, Nathalie Arbour, Thierry Alquier, Stephanie Fulton

**Affiliations:** 1 Centre de Recherche du Centre Hospitalier de l’Université de Montréal (CRCHUM), Montréal, QC H3T1J4 Canada; 2 Department of Nutrition, Université de Montréal, Montréal, QC H3T1J4 Canada; 3 Department of Medicine, Université de Montréal, Montréal, QC H3T1J4 Canada; 4 Department of Neuroscience, Université de Montréal, Montréal, QC H3T1J4 Canada; 5 Nutrition and Integrative Neurobiology Unit, UMR 1286, INRA-Université de Bordeaux, Bordeaux, France; 6 Present Address: Research Group in Cellular Signaling, Department of Medical Biology, Université du Québec à Trois-Rivières, Trois-Rivières, Canada

**Keywords:** Free fatty acid receptor, Ffar4, Endotoxemia, Nucleus accumbens, Anxiety, Sickness behaviors, Locomotion, Neuropharmacology, Behavioral neuroimmunology

## Abstract

G protein-coupled receptor 120 (GPR120, Ffar4) is a sensor for long-chain fatty acids including omega-3 polyunsaturated fatty acids (n-3 PUFAs) known for beneficial effects on inflammation, metabolism, and mood. GPR120 mediates the anti-inflammatory and insulin-sensitizing effects of n-3 PUFAs in peripheral tissues. The aim of this study was to determine the impact of GPR120 stimulation on microglial reactivity, neuroinflammation and sickness- and anxiety-like behaviors by acute proinflammatory insults. We found GPR120 mRNA to be enriched in  both murine and human microglia, and in situ hybridization revealed GPR120 expression in microglia of the nucleus accumbens (NAc) in mice. In a manner similar to or exceeding n-3 PUFAs, GPR120 agonism (Compound A, CpdA) strongly attenuated lipopolysaccharide (LPS)-induced proinflammatory marker expression in primary mouse microglia, including tumor necrosis factor-α (TNF-α) and interleukin-1β (IL-1β), and inhibited nuclear factor-ĸB translocation to the nucleus. Central administration of CpdA to adult mice blunted LPS-induced hypolocomotion and anxiety-like behavior and reduced TNF-α, IL-1β and IBA-1 (microglia marker) mRNA in the NAc, a brain region modulating anxiety and motivation and implicated in neuroinflammation-induced mood deficits. GPR120 agonist pre-treatment attenuated NAc microglia reactivity and alleviated sickness-like behaviors elicited by central injection TNF-α and IL-1β. These findings suggest that microglial GPR120 contributes to neuroimmune regulation and behavioral changes in response to acute infection and elevated brain cytokines. GPR120 may participate in the protective action of n-3 PUFAs at the neural and behavioral level and offers potential as treatment target for neuroinflammatory conditions.

## Highlights

- GPR120 is enriched in murine and human microglia.
- Central GPR120 agonism decreases microglia activation and anxiety- and sickness-like behaviors in response to LPS.
- GPR120 agonism attenuates nucleus accumbens microglia reactivity and behavioral responses to central cytokine administration.

## Introduction

Neuroinflammation emanating from systemic immune activation contributes to mood and emotional disturbances and sickness-like behaviors like psychomotor slowing. Microglia play a central role neuroimmunity and their activation can be triggered by immunometabolic responses provoked by poor diet and obesity [1, 2]. Several reports demonstrate that high-fat feeding or administration of lipopolysaccharide (LPS), a gut-derived bacterial toxin upregulated by a high-fat diet, provokes microglial reactivity and cytokine expression in various brain regions including the hypothalamus, hippocampus, and nucleus accumbens (NAc) [3–5]. The NAc regulates motivated behavior and mood states and is well-implicated in the pathophysiology of anxiety and depression [6]. We previously found that a saturated, but not a monounsaturated, high-fat diet triggers glial reactivity and inflammatory indices in the NAc that contribute to anxiety- and depressive-like behaviors in mice, effects prevented by a local viral intervention blocking nuclear factor-ĸB (NFκB) activation [4].

Omega-3 polyunsaturated fatty acids (n-3 PUFAs) have central actions that mitigate neuroinflammation in part by suppressing microglial cytokine and chemokine production. Dietary n-3 PUFAs supplementation protects against the proinflammatory effects of LPS, diet-induced obesity, neural injury, and chronic stress in peripheral tissues and brain [7, 8]. G-protein coupled receptor 120 (GPR120), also known as free fatty acid receptor 4 (FFAR4), mediates actions of n-3 PUFAs such as α-linolenic acid (ALA; 18:3n-3), eicosapentaenoic acid (EPA; 20:5n-3), and docosahexaenoic acid (DHA; 22:6n-3), on gut hormone secretion and systemic inflammation [9, 10]. Peripheral GPR120 is expressed in the intestine and lung but is particularly enriched in immune cells. Pharmacological GPR120 stimulation was shown to suppress macrophage-mediated adipose tissue inflammation and improve insulin resistance through the inhibition of NFκB [11]. GPR120 is also expressed in the brain [12, 13], and we previously found that intracerebroventricular (ICV) GPR120 agonist infusion suppress feeding, food reward and anxiety-like behavior caused by a saturated high-fat diet [12]. Despite these results, the contribution of GPR120 activation to microglial reactivity, neuroinflammation and associated behavioral deficits in response to neuroimmune challenges is unclear. The present study set out to determine the GPR120 neural expression profile in mouse and human and NAc murine microglia; the capacity of GPR120 agonism to moderate acute proinflammatory responses in microglia, including NAc microglia, and the influence of central GPR120 agonism on anxiety- and sickness-like behaviors and neuroinflammatory indices in the NAc produced by acute systemic (LPS) and central (cytokines) inflammatory interventions.

## Materials and methods

### Animals

All experiments were approved by the Institutional Animal Care Committee of the Centre de Recherche du Centre Hospitalier de l’Université de Montréal (CRCHUM) in accordance with the standards of the Canadian Council on Animal Care. Eight to ten-week-old C57Bl/6J male mice from Jackson Laboratories (Bar Harbor, Maine, USA) were used for gene expression and behavioral tests. All animals were maintained in an environmentally controlled room (22–24 ºC) on reverse light/dark cycle (light phase 10:00 pm to 10:00 am) with ad libitum access to standard chow and water.

### Chemicals and reagents

LPS from *Escherichia coli* (L-4516, serotype 0127:B8), oleic acid (OA; O1008), alpha-linolenic acid (ALA, L2376), eicosapentaenoic acid (EPA, E2011), and docosahexaenoic acid (DHA, D2534) were purchased from Sigma–Aldrich (St. Louis, MO, USA) and aliquoted for single freeze–thaw use. Mouse recombinant TNF-α (410-MT-010/CF) and IL-1β (401-ML-010/CF) were purchased from R&D systems Inc. (Minneapolis, MN, USA). Papain and DNase I were purchased from Worthington Biochemical corp. (Lakewood, NJ, USA). Compound A (CpdA) was purchased from Cayman Chemical (Ann Arbor, MI, USA). Cell culture reagents were purchased from ThermoFisher Scientific (Waltham, MA, USA) unless specified.

### Murine neural cells

Primary microglia were prepared from whole forebrain or NAc microdissections of C57BL/6 pups at PND 1–3 [14]. Briefly, cell suspensions were treated with an enzymatic solution containing papain (9 U/ml), DNase (200 U/ml), glucose (5 mg/ml), cysteine (0.2 mg/ml), and bovine serum albumin (0.2 mg/ml) for 15 min at 37 °C in 5% CO_2_. Debris were removed by the passing with 70 μm cell strainer. Mixed glial cells were cultured in T75 flask and maintained in Dulbecco’s modified Eagle’s medium (DMEM) and supplemented with 10% heat-inactivated fetal bovine serum (FBS) and 1% of antibiotics (penicillin G (10,000 U/ml)-streptomycin sulfate (10,000 μg/ml) at 37 °C in 5% CO_2_. Media were replaced at day 7 in vitro and culture was maintained until reaching astrocyte confluence (10–14 days). Subsequently, microglia grow on top of a single layer of astrocytes. The culture medium containing primary microglia was transferred to poly-_L_-lysine (PLL)-coated 12 mm coverslips or 24-well plates for a maximum of five days prior to treatment. To evaluate the expression of GPR120 in different neural cell types, primary astrocytes and neurons derived from the procedures above were also cultured. Primary neurons were cultured in PLL-coated 24-well plates in Neurobasal-A medium containing, 2% B-27 supplement, 1% Glutamax, and 1% antibiotics solution for 7 days in vitro.

### Human neural cells

Fetal brain tissue (17–21 weeks) was obtained following informed consent (University of Washington, Seattle, Washington, USA, STUDY00000380) and experiments were approved by the CRCHUM ethics boards (BH07.001, HD07.002). Astrocytes and microglia were isolated from fetal brain tissue as previously described [15, 16] and cultured in DMEM containing 10% FBS. Neurons were collected as negative fraction after removal of MHC class I (glial cells) and CD235a (red blood cells) expressing cells using Miltenyi beads. Cells were cultured in DMEM F12 without red phenol containing 2% MACS^®^ NeuroBrew^®^ (Miltenyi Biotec), penicillin 100U/mL, streptomycin 100 µg/mL, and 20 mM HEPES [16].

### Culture treatments

Free fatty acids (FFA) (OA, ALA, EPA, and DHA) and CpdA were dissolved in ethanol for stock solution at 100 mM and diluted in the culture media to a final concentration of 10 μM in 0.1% ethanol. FFA concentration was selected according to our previous studies [17]. The CpdA dose selected for culture experiments was based on that used in cultured macrophages [11]. LPS was dissolved in phosphate-buffered saline (PBS) and diluted to a final concentration of 100 ng/mL. Primary microglia were cultured in the DMEM without FBS and antibiotic for 24 h before treatment. Serum-free medium was used for FFA and CpdA application. Microglial cells were pre-treated for 1 h with FFAs or CpdA (10 μM) before adding LPS (100 ng/ml) or the cytokine mixture (TNF-α + IL-1β, 50 ng/ml). After 6 h incubation, supernatants were harvested for ELISA and cells were processed for RNA extraction.

### Stereotaxic surgery

Mice were individually housed one week prior to ICV cannula implantation. Animals were anesthetized with isoflurane (3% induction; 1–2% maintenance) and positioned in an Ultraprecise Mouse stereotaxic apparatus (Kopf Instruments). A single ICV guide cannula (C315GS-5-SP, 5 mm, 26 gauge, Plastics One) was implanted into the right cerebral ventricle using stereotaxic coordinates (+ 0.5 mm caudal and + 1 mm lateral; − 2.0 mm ventral from dura). The cannula was secured to the skull with cyanoacrylate glue and dental cement and closed with an adapted dust cap (Dummy cannula: C315DCS-5-SPC, 5 mm, Plastics One). Correct positioning of the cannula was verified seven days after surgery by the drinking response elicited by injection of angiotensin II (20 ng/μL; Sigma). For histochemical verification, mice were anesthetized by intraperitoneal (IP) pentobarbital injection and then were perfused with cold PBS and 10% neutral buffered formalin. Brains were post-fixed with 10% neutral buffered formalin overnight followed by an increasing sucrose gradient.

### Procedures for behavioral assessment

To assess the behavioral effects of central GPR120 agonism in the context of acute inflammation, mice received ICV CpdA (10 μg in 2 µl 16% DMSO) or vehicle daily during three consecutive days. On day three, one cohort of mice was euthanized 2 h following LPS (0.83 mg/kg, IP) injection for gene expression studies whereas behavioral testing was carried out 12 h after intraperitoneal LPS or ICV cytokine mixture (each 50 ng in 2 µl 0.2% BSA in saline; end of light cycle) in a separate cohort. LPS dose was chosen based on previous our work which confirmed increase of cytokine levels in plasma [18]. The CpdA dose was based on Oh et al. and adapted for ICV administration [11, 19]. Control mice received an IP injection of vehicle (endotoxin-free saline solution). Cytokine doses chosen were based on reports of the minimal effective dose to elicit anxiety- and depressive-like behavior [20–22].

### Elevated-plus maze

The elevated-plus maze (EPM) served as the first test of sickness and anxiety-like behavior and was performed as reported [23]. Briefly, each mouse was placed in the center of the maze facing an open arm opposing the experimenter. Distance travelled, the proportion of time spent in the open arms, and the number of entries to the open arms were measured by an overhead video camera connected to a PC with Ethovision XT software (Med Associates, Inc.) for a period of five minutes.

### Light/dark box task

The light/dark box (LDB) was employed as a secondary test of sickness and anxiety-like behavior. The apparatus (Med Associates, Inc.) consists of an illuminated compartment of transparent plastic walls and a dark compartment with black walls, covered by a lid (both 13.7 cm × 13.7 cm × 20.3 cm). The two boxes are separated by a partition wall, with an opening at the bottom to allow the animal to pass freely between compartments. Number of entries and time spent in the lit compartment of the box were measured by an overhead video camera connected to a PC with Ethovision XT software (Med Associates, Inc.) for a period of five minutes.

## Three-chamber social interaction test

The three-chamber social interaction test (3CT) was used to assess general sociability and interest in social novelty as an inference of anxiodepressive behavior. The rectangular apparatus (40 cm × 60 cm × 23 cm) contains three connected compartments divided by opaque Plexiglas walls. An unfamiliar male stimulus mouse was placed under an open-wire cup on one side of the chamber, whereas an empty wire cup was placed on the opposing side. Experimental mice were introduced to the chamber center. Distance travelled, the proportion of time spent, the number of entries in the stimulus chamber and the average time spent in the stimulus mouse zone were analyzed by an overhead video camera connected to a PC with Ethovision XT software (Med Associates, Inc.) for a period of five minutes.

### Quantitative PCR

For cell culture experiments, TRIzol (Invitrogen) was directly added to wells. Following in vivo experiments, frozen brains were sliced with a cryostat, brain nuclei collected by tissue punch and RNA extracted using TRIzol. RNA concentration was quantified, and 1000 ng of total RNA was reverse-transcribed by M-MuLV reverse transcriptase (Invitrogen) with random hexamers following protocol. Quantitative gene expression was measured from 1:5 cDNA dilutions. RT-qPCR were performed using the QuantiFast SYBR Green PCR kit (Qiagen, Valencia, CA, USA) according to the manufacturer's guidelines on a Corbett Rotor-Gene 6000. Quantitative real-time PCR for GPR120 (*Ffar4*), interleukin 1 beta (IL-1β), interleukin 6 (IL-6), tumor necrosis factor-alpha (TNF-α), C–C motif chemokine ligand 2 (CCL2), ionized calcium binding adaptor molecule 1 (Iba-1), and 18S (reference gene) were carried out using specific primers (sequences in Table 1). PCRs were performed in triplicate and relative gene expression was calculated using the ΔΔCT method using BACT (for human) or 18S (for mouse) as housekeeping genes.

**Table 1:** A table of the primer sets used in qRT-PCR

Gene	Forward primer (5′–3′)	Reverse primer (3′–5′)
h-BACT	TGA CGG GGT CAC CCA CAC TGT GCC CAT CTA	CTA GAA GCA TTT GCG GTG GAC GAT GGA
m-18S	TAG CCA GGT TCT GGC CAA CGG	AAG GCC CCA AAA GTG GCG CA
h-FFAR4	TGG AGA TGC ACA TTG TTT GGA GA	AGC CTC CAA GTG GTG GAG TGA
m-Ffar4	TTT ACA GAT CAC GAA AGC ATC GC	GTG CGG AAG AGT CGG TAG TC
m-Iba-1	GGA TTT GCA GGG AGG AAA AG	TGG GAT CAT CGA GGA ATT G
m-IL-1β	GAC CCC AAA AGA TGA AGG GCT	ATG TGC TGC TGC GAG ATT TG
m-IL-6	CAG AGT CCT TCA GAG AGA TAC	AGC TTA TCT GTT AGG AGA GC
m-TNF-α	CAC GCT CTT CTG TCT ACT G	AAG ATG ATC TGA GTC TGA GG
m-Ccl2	ATT GGG ATC ATC TTG CTG GT	CCT GCT GTT CAC AGT TGC C

### Cytokine release

After treatment, microglial cell culture media were collected and immediately frozen. Murine TNF-α, IL-1β, IL-6 and CCL2 was measured using the antibodies and reference standards contained in R&D Systems (Minneapolis, MN, USA) enzyme-linked immunoabsorbent assay (ELISA) Duokits according to the manufacturer’s protocol.

### Immunochemistry

Microglia cultures were fixed with fresh 4% paraformaldehyde. Primary antibody for rabbit anti-IBA-1 (1:1000, FUJIFILM Wako Chemicals U.S.A. Co., VA) or rabbit anti-NFκB (p65) (1:250, Santa Cruz Biotechnology, Inc., TX) in blocking solution (0.2% Triton-X 100 and 10% normal goat serum in PBS) was applied overnight at 4 °C. After washing with PBS several times, secondary antibody (goat anti-mouse IgG Alexa568 or goat anti-rabbit IgG Alexa488, 1:500) in blocking solution was applied for 1 h at room temperature. Slide-mounted blain slices (14 µm) were treated with EDTA (pH 6.0) and boiled for 10 min for antigen retrieval [24]. Slices were incubated with a blocking solution (0.3% Triton-X 100 and 3% normal goat serum in PBS) for 1 h. Primary antibody for rabbit anti-IBA-1 (1:500) was applied to brain slice overnight at 4 °C. After washing with PBS several times, secondary antibody (Goat anti-rabbit IgG Alexa488, 1:500) in blocking solution was applied for 2 h at room temperature. Cell cultures and brain slices were washed with PBS followed by the application of mounting media containing Dapi (Vectashield, Vector Laboratories, Inc., Newark, CA, USA). Z-stack images were captured with a Zeiss AxioImager 2 (Carl Zeiss AG, Jena, Germany) and analyzed with ImageJ/Fiji. For morphological analysis, the 16-bit images were converted to binary images after determining threshold at equivalent levels for all samples. Noise reduction by despeckle and fix cell shape by -close command were performed [25]. Cell length, area, and circularity were assessed. For NFκB analysis, the intensity of nuclear and cytoplasmic signals was measured followed by calculating the ratio of nuclear-to-cytoplasmic area.

### In situ hybridization (RNAScope^®^)

Slide-mounted brain slices (14 µm) were baked at 60 °C for 30 min. Slices were dehydrated with ethanol and endogenous peroxidase action was removed by a 5 min H_2_O_2_ treatment. Tissues were boiled in antigen retrieval reagent for 15 min, and then digested with protease III at 40 °C for 30 min in the HybEZ™ II Oven (ACD Bio). The detection of mouse Ffar4 (ACD Bio. Cat. 447,041) and mouse Tmem119 (ACD Bio. Cat. 472,901-C2) mRNA expression in NAc was performed with RNAscope Multiplex Fluorescent V2 Assay according to manufacturer’s protocol. The Z-stack images were captured with Zeiss AxioImager 2 (Carl Zeiss AG, Jena, Germany) and processed with ImageJ/Fiji.

### ***Statistical ***analyses

All data are expressed as mean ± SEM. Data were analyzed using GraphPad Prism 9 (San Diego, CA, USA). Between-group comparisons were made with a one-way ANOVA with Sidak post hoc tests. Criteria for statistical significance were set at *p* ≤ 0.05.

## Results

### GPR120 activation attenuated LPS-induced cytokine production in primary microglia

**Fig. 1:**
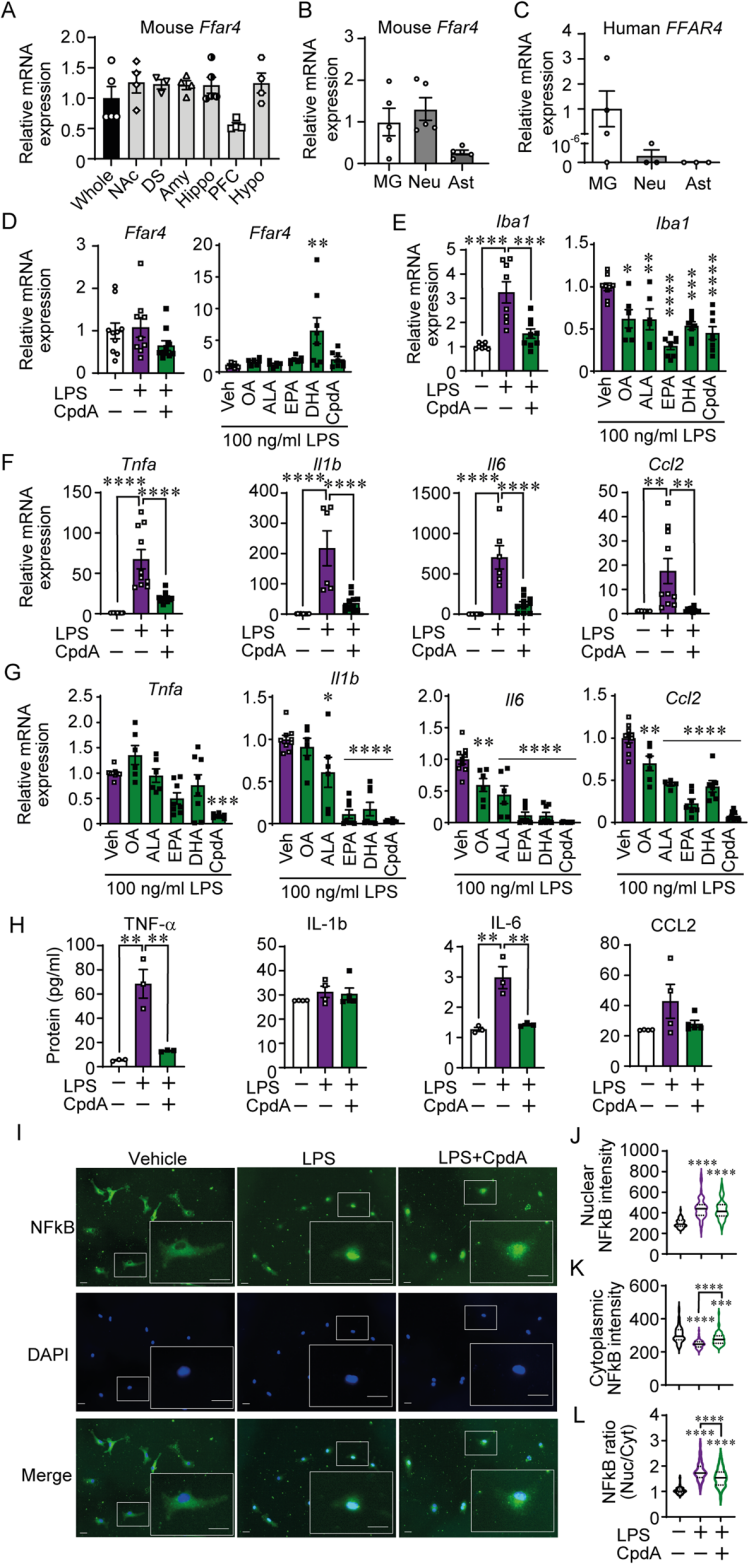
Central GPR120 expression and microglial anti-inflammatory function. **A** Distribution of GPR120 (free fatty acid receptor 4, Ffar4) mRNA in mouse brain (whole: whole brain, NAc: nucleus accumbens, DS: dorsal striatum, Amy: amygdala, Hippo: hippocampus, PFC: prefrontal cortex, Hypo; hypothalamus) (*n =* 3–5/group). **B** GPR120 mRNA expression in primary murine microglia (MG), neurons (Neu), and astrocytes (Ast) (*n =* 5). **C** GPR120 mRNA expression in human fetal microglia (MG), neurons (Neu), and astrocytes (Ast) (*n =* 3–4). Effect of GPR120 agonism on **D** Ffar4 and **E** Iba-1 mRNA expression on LPS-stimulated primary microglia (*n =* 6–11/group). Proinflammatory cytokine mRNA expression on LPS-stimulated primary cultured microglia pre-treated with **F** CpdA or **G** unsaturated FAs (OA; oleic acid, ALA; α-linolenic acid, EPA; eicosapentaenoic acid, DHA; docosahexaenoic acid). **H** Cytokine protein levels in culture medium (*n =* 3–5/group). **I** Representative image of NFκB translocation after LPS-treatment with or without CpdA pre-treatment. Scale bar, 20 µm. NFκB intensity in **J** nuclear and **K** cytoplasmic compartments. **L** Ratio of nuclear/cytoplasmic NFκB (*n =* 63, from 3 cover slips). Data are expressed as the mean ± SEM. One-way ANOVA with post hoc Sidak multiple comparison test; *p ≤ 0.05, **p ≤ 0.01, ***p ≤ 0.001, ****p ≤ 0.0001 vs Veh LPS

We first set out to determine the gene expression profile of GPR120 in different regions of the mouse brain. Similar levels of GPR120 mRNA were expressed in the NAc, dorsal striatum (DS), amygdala (Amy), hippocampus (Hippo) and hypothalamus, with lower expression in the prefrontal cortex (PFC) (Fig. 1A). In cultured mouse brain cells, we found relatively higher expression of GPR120 mRNA in microglia and neurons as compared to astrocytes (Fig. 1B). A similarly higher level of GPR120 mRNA was found in human fetal microglia relative to human astrocytes and neurons (Fig. 1C). GPR120 levels were not affected by LPS and/or GPR120 agonist (CpdA) application in primary murine microglia; however, DHA application increased GPR120 mRNA in LPS-treated microglia (Fig. 1D).

To study the role of microglial GPR120, we evaluated GPR120 agonism in the context of an endotoxin challenge. Pretreatment with CpdA blunted LPS-induced Iba-1 mRNA (microglial reactivity marker) expression in a manner similar to n-3 and n-9 unsaturated FFA (Fig. 1E). GPR120 agonism markedly reduced LPS-induced expression of TNF-α, IL-1β, IL-6, and Ccl2 (Fig. 1F). DHA and EPA showed a stronger anti-inflammatory effect compared to OA (a n-9 fatty acid) in LPS-treated microglia (Fig. 1G). These changes were accompanied by reduced secretion of TNF-α and IL-6 protein levels in the culture medium by CpdA (Fig. 1H). NFκB is a key factor upregulating cytokine and chemokines that upon phosphorylation translocates to the nucleus to regulate gene transcription [26]. LPS increased NFκB nuclear localization (Fig. 1I, J); however, CpdA pre-treatment partially prevented this (Fig. 1K) and reduced the ratio of nuclear/cytoplasmic NFκB (Fig. 1L).

### Central GPR120 agonism attenuated LPS sickness- and anxiety-like behaviors and inflammatory indices in the nucleus accumbens

**Fig. 2:**
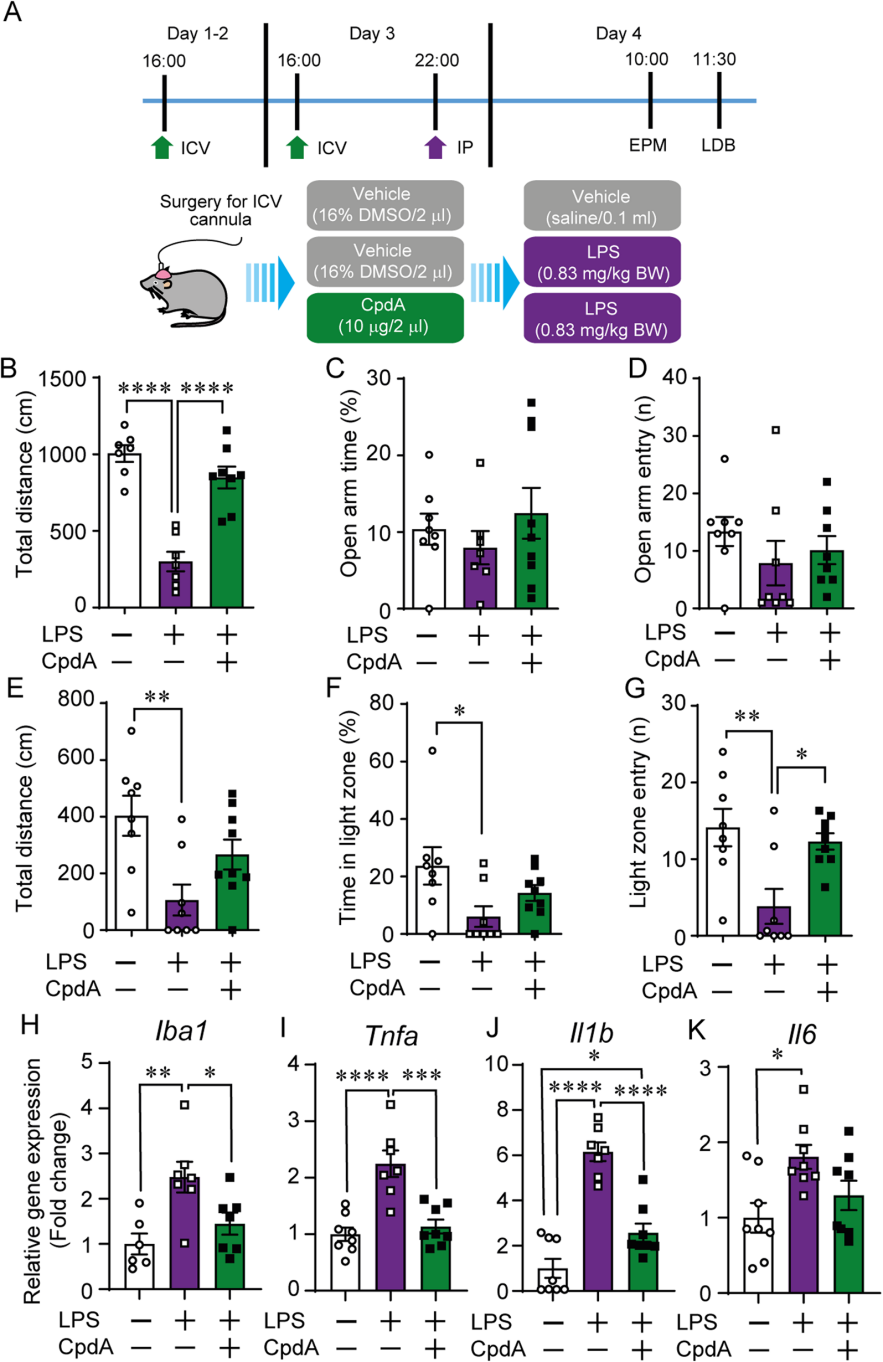
Central GPR120 agonism reduces LPS-induced hypolocomotion and anxiety-like behavior. **A** Experimental design of LPS-CpdA in vivo study 1. **B** Total distance travelled, **C** time spent, and **D** number of entries made into open arms of the elevated-plus maze (EPM). **E** Total distance travelled, **F** time spent, and **G** number of entries in the light compartment of the light–dark box. Effect of GPR120 agonism on LPS-induced **H** Iba-1, **I** TNF-α, **J** IL-1β, and **K** IL-6 mRNA expression in the nucleus accumbens (NAc). *n =* 7–8/group. Mean ± SEM; *p ≤ 0.05, **p ≤ 0.01, ****p ≤ 0.0001

We next evaluated if ICV administration of CpdA could abrogate LPS-induced decreases in locomotion and anxiety-like behavior using two well-established behavioral tests (Fig. 2A). Three days of ICV CpdA pre-treatment blocked systemic LPS-induced hypolocomotion (distance travelled) in the EPM test (Fig. 2B); however, LPS and CpdA did not significantly alter the proportion of time spent (Fig. 2C) and the number of entries in the open arms (Fig. 2D). Mice also demonstrated anxiety-like behaviors in the light–dark box task following LPS injection as shown by a reduction of the total distance traveled, time spent and number of entries in the lit compartment (Fig. 2E–G). All of these effects were blunted by CpdA suggesting that central GPR120 activation protects against the anxiogenic and psychomotor slowing effects elicited by acute systemic immune activation (Fig. 2E–G).

As there is heterogeneity in microglia reactivity across brain regions [26], we also investigated the capacity of GPR120 stimulation to alleviate inflammation in the NAc, a region in which inflammatory insults affect locomotor activity, anxiodepressive behavior and increased food-motivated behavior [4, 28]. In a manner resembling results in primary microglia, expression of the microglial marker Iba-1 in the NAc was increased by LPS-injection and attenuated by CpdA pre-treatment (Fig. 2H). Furthermore, in vivo CpdA pre-treatment significantly prevented LPS-induced increases in NAc TNF-α and IL-1β (Fig. 2I–K).

### Anti-inflammatory actions of GPR120 activation in nucleus accumbens microglia

**Fig. 3:**
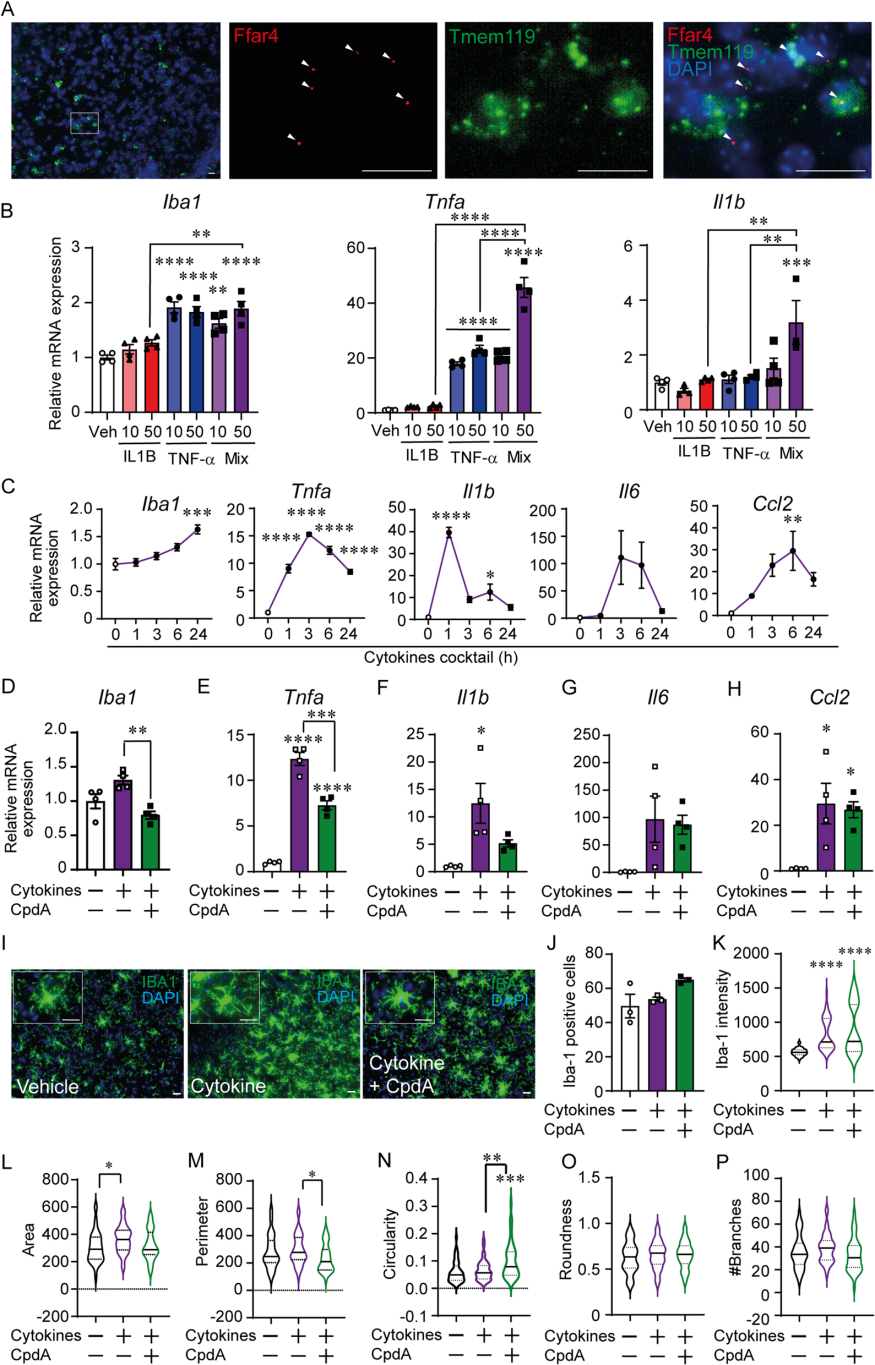
GPR120 agonism attenuates nucleus accumbens microglial reactivity. **A** GPR120 (red) expression in NAc microglia (Tmem119, green) detected by RNAscope in situ hybridization in adult mice. Scale bar, 20 µm. **B** The effect of IL-1β and/or TNF-α application on Iba-1, TNF-α, and IL-1β mRNA expression in primary microglia derived from NAc. **C** Time course of cytokine -induced proinflammatory and microglial marker mRNA expression. Effect of GPR120 agonist pre-treatment on cytokine-induced **D** Iba-1, **E** TNF-α, **F** IL-1β, **G** IL-6, and **H** Ccl2 mRNA expression (*n =* 4). **I** Representative images of NAc microglia in slice preparations derived from mice ICV-injected with the TNF-α and IL-1β mixture with/without ICV CpdA pre-treatment. Scale bar, 20 µm. **J** Number of Iba-1 positive cells (*n =* 3). **K** Iba-1 intensity, **L** cell area, **M** cell perimeter, **N** circularity, **O** roundness, and **P** branches (*n =* 41–47 microglia/*n =* 3 brain). Mean ± SEM; one-way ANOVA with post hoc Sidak multiple comparison test; *p ≤ 0.05, **p ≤ 0.01, ***p ≤ 0.001, ****p ≤ 0.0001 vs Veh

Increasing proinflammatory cytokine levels specifically in the brain also triggers neuroinflammation and behavioral perturbations. Neuroinflammatory responses in the NAc have been linked to reduced movement and enhanced anxiodepressive behavior [4, 28]. Thus, we first sought to examine NAc GPR120 expression and if GPR120 stimulation protects against the activation of NAc microglia in response to central of TNF-α and IL-1β—two cytokines required for the innate immune response, that influence affective states and that are upregulated in the brain in response to systemic LPS, stress and obesity [2, 29]. First, RNAscope i*n situ* hybridization revealed GPR120 co-expression with the microglial marker Tmem119 in the NAc (Fig. 3A). Co-treatment with TNF-α and IL-1β increased Iba-1, TNF-α, and IL-1β mRNA expression in primary NAc microglia more so than either cytokine alone (Fig. 3B). The cytokine mix increased proinflammatory cytokine expression in a time- and cytokine-dependent manner (Fig. 3C). CpdA attenuated increases in Iba-1, TNF-α, and IL-1β mRNA expression (Fig. 3D–F) without affecting IL-6 and Ccl2 expression (Fig. 3G–H). Next, we assessed NAc microglial morphology in slices derived from mice treated with the ICV cytokine mix with or without 3-day CpdA pre-treatment (Fig. 3I). The number of IBA-1 positive cells did not significantly change (Fig. 3J), however Iba-1 intensity was significantly increased by ICV cytokine mix with or without CpdA pre-treatment (Fig. 3K). While, microglial cell area was greater in cytokine condition, an effect was prevented by CpdA (Fig. 3L). Moreover, CpdA reduced cell perimeter (Fig. 3M) and increased cell circularity compared to vehicle and cytokines alone (Fig. 3N), whereas there was no significant difference among the group in roundness (Fig. 3O) and branches (Fig. 3P).

### Central GPR120 agonism attenuated sickness-like behaviors by cytokines

**Fig. 4:**
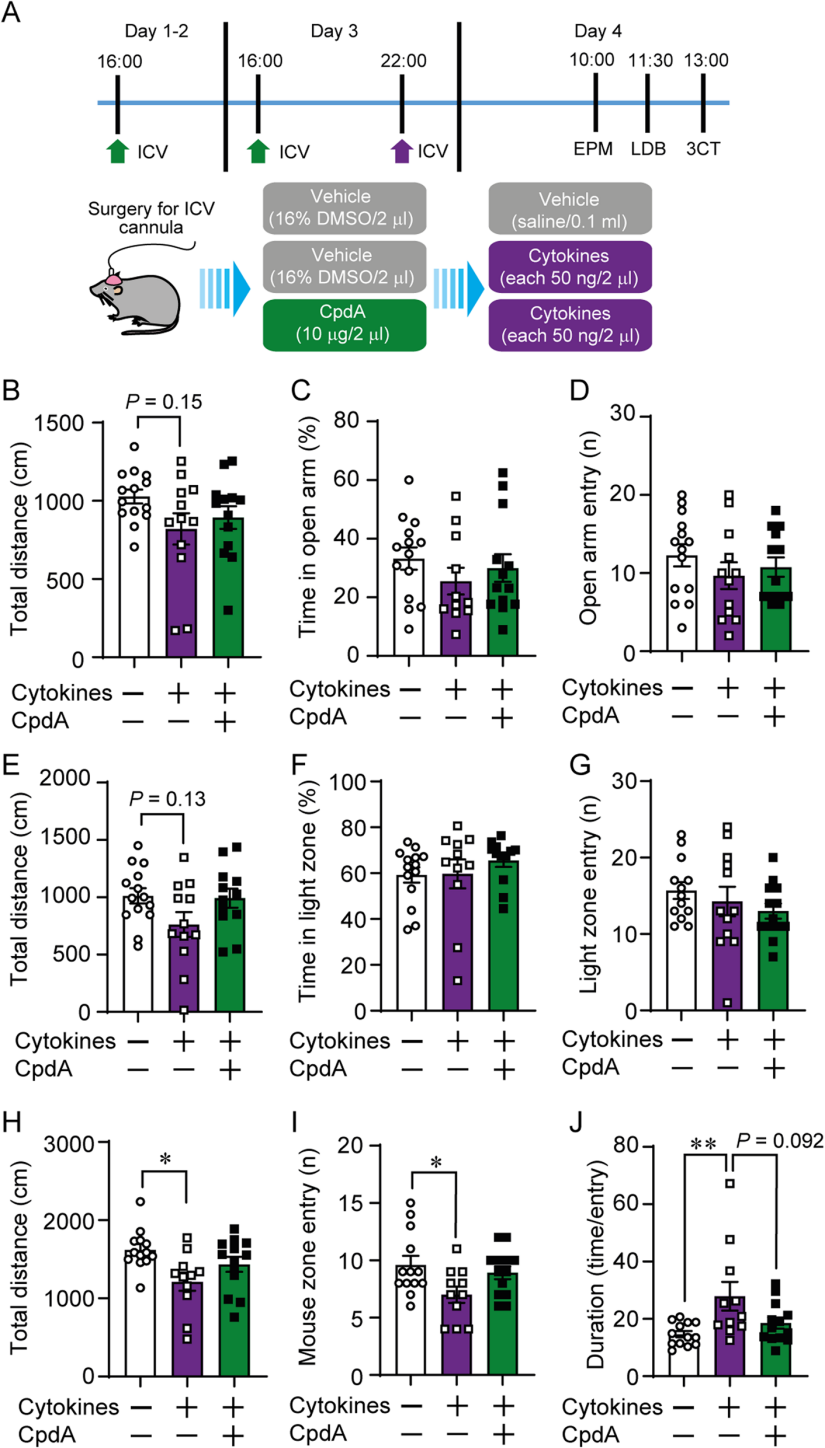
Central GPR120 agonism protects against central cytokine-induced behavioral responses. **A** Experimental design of central cytokine in vivo study 2. **B** Total distance travelled, **C** time spent, and **D** number of entries made in open arms of the elevated-plus maze (EPM) test. **E** Total distance travelled, **F** time spent, and **G** number of entries made in the light compartment of the light–dark box (LDB) test. **H** Total distance travelled, **I** number of entries in mouse zone, **J** duration of time spent in mouse zone in the 3-chamber (3-CT) social interaction test. *n =* 11–14/group. Mean ± SEM; one-way ANOVA with post hoc Sidak multiple comparison test;*p ≤ 0.05, **p ≤ 0.01

We next examined if ICV CpdA could diminish anxiety- and sickness-like behaviors produced by central administration of the cytokine mix (Fig. 4A). There was a trend for reduced locomotor activity following ICV cytokine administration in the EPM (*P* = 0.15, Fig. 4B) and LDB (*P* = 0.13, Fig. 4E), however, anxiety-like behavior as inferred by proportion of time spent in open arms or light box was unchanged in the EPM (Fig. 4C, D) and LDB (Fig. 4F, G), respectively. We next submitted mice to a social interaction test. Central cytokine injection reduced total distance travelled in the 3CT and this effect was prevented in mice pre-treated with CpdA (Fig. 4H). Cytokine treatment also reduced the number of entries in the stimulus mouse zone (Fig. 4I) and the average duration (time in mouse zone/entry number) (Fig. 4J) which were both prevented by CpdA treatment.

## Discussion

**Fig. 5:**
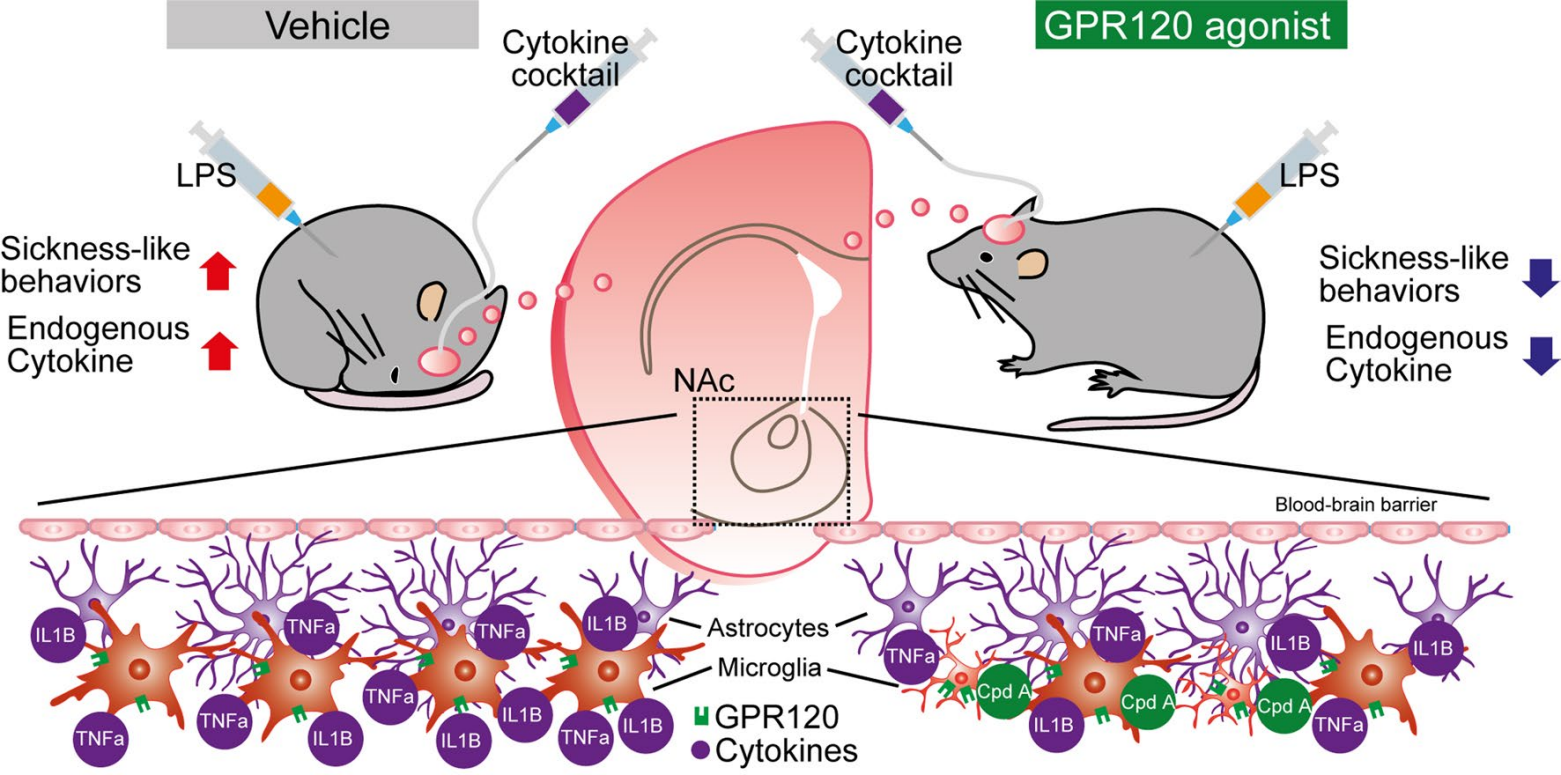
Working model of GPR120 stimulation modulation of acute neuroinflammation and associated behavioral changes. GPR120 agonism attenuates LPS or cytokines-induced microglial activation and cytokine production. Sickness- or anxiety-like behaviors caused by systemic LPS injection and central cytokine infusion are rescued by ICV GPR120 agonist treatment

The present study demonstrates that GPR120 is relatively enriched in microglial neural cells in both mouse and human and that its activation in murine microglia can significantly blunt acute neuroimmune responses. GPR120 agonism abrogated LPS-induced inflammation in primary microglia in a manner that resembled or exceeded the effects of n-3 fatty acids. Further, central GPR120 agonist pre-treatment was effective in attenuating sickness- and anxiety-like behaviors triggered by systemic LPS and central TNF-α and IL-1β administration. Together, these results provide evidence that activation of microglial GPR120 serves to mitigate neuroinflammation and suggest that intake of n-3 fatty acids or treatment with GPR120-based therapies that enter the CNS could offer a successful means to alleviate neuroinflammatory conditions (Fig. 5).

PUFAs and their metabolites including eicosanoids and prostaglandins are immune modulators in peripheral tissues and the CNS [30–32]. However, the receptor signaling pathways involved in FFA modulation of neuroinflammation remain elusive. Our observations that GPR120 is localized to murine and human microglia agree with microglia RNA sequencing findings [33]. Moreover, the present results corroborate and broaden the neuroprotective properties of GPR120 implied by results showing GPR120-positive microglia increase after focal cerebral ischemic injury and that GPR120 activation by DHA rescues ischemic injury and neuroinflammation induced by the Japanese encephalitis virus in mice [34, 35]. Revealing further benefits of microglial GPR120 stimulation, we discovered that GPR120 agonism strongly diminishes LPS-induced cytokine expression and secretion in primary microglia in a manner similar or greater than the well-known effects of n-3 PUFA application. These actions were mediated by reduced NFκB nuclear translocation in microglia by LPS, consistent with its anti-inflammatory signaling actions in peripheral macrophages [11].

As there is heterogeneity in microglial gene expression and activity patterns between different brain areas [27] and we are exploring pharmacological strategies to dampen inflammation in structures controlling mood and motivation, we also investigated the influence of GPR120 stimulation in microglia derived from the NAc. Inflammatory insults in the NAc contribute to reduced locomotor activity, anxiodepressive behavior and increased food-motivated behavior [4, 28]. We found that the beneficial actions of GPR120 agonism also extended to the NAc microglia reactivity in response to both systemic LPS and combined TNF-α and IL-1β application. Thus, the effects of n-3 supplementation to prevent saturated high-fat diet induced increases in glial reactivity in the NAc [8] could be partly mediated by GPR120 activation. These findings are consistent with data demonstrating that blockade of NFκB- and TNF-α-mediated inflammatory responses in the NAc recovers anxiodepressive behaviors in diet-induced obese and diabetic mice, respectively [4, 36]. Correspondingly, we evaluated behaviors that involve NAc neurotransmission and neuroplasticity to again reveal a protective influence of central GPR120 agonism. Suppression of locomotor activity caused by LPS was blocked by GPR120 agonist pre-treatment in two tasks. Behavioral changes typically characterizing anxiolytic actions were only observed in the light–dark box tests after LPS, whereas the effects of GPR120 activation to attenuate psychomotor slowing were consistently observed. The lack of differences in open-arm time in the elevated-plus maze task could be due to strong locomotor deficits that could have caused highly variable time spent in the open arm. The alleviation of sickness-like behaviors by GPR120 agonist pre-treatment coincided with a considerable reduction in proinflammatory marker (Iba-1, TNF-α, IL-1β, and IL-6) expression in the NAc. Elevated IL-1β and TNF-α expression in the brain is one of the hallmarks of rodent depressive-like behavior by LPS [37, 38]. In addition, ICV injection of these proinflammatory cytokines induces sickness and anxiodepressive behavior [22, 39, 40].

Here, we found that mouse, but not human neurons also expressed GPR120 at a level similar to microglia. Neuronal GPR120 expression was also observed in the hippocampus and cortex of the mouse brain, regions where virally mediated GPR120 overexpression reduced epileptic seizure activity and neuroinflammation in a mouse model of epilepsy [41]. GPR120 agonist and DHA also attenuated TNF-α-induced inflammation in immortalized hypothalamic mouse neurons [42]. Together, these studies suggest that the GPR120 agonist could directly act on neurons to reduce neuronal inflammatory responses. NAc microglia-derived TNF-α altered the activity of D1-expressing medium spiny neurons and cocaine-induced behavior sensitization [28]. These findings raise the possibility that GPR120 agonism may indirectly modulate dopamine neuron activity by reducing microglial proinflammatory cytokine secretion. Addressing these possibilities would require additional studies to identify the cell type(s) in the mesoaccumbal circuit (e.g., NAc and ventral tegmental area) expressing GPR120. Finally, based on the expression pattern of GPR120 in peripheral epithelial cells and macrophages [11], we cannot rule out the possibility that the GPR120 agonist also targets other cell types in the brain including those composing the blood–cerebrospinal fluid barrier (e.g., endothelial and choroid epithelial cells, perivascular macrophage, or pericytes) to reduce inflammatory responses.

While combined TNF-α and IL-1β administration failed to significantly modulate sickness- and anxiety-like behaviors in the EPM and LDB tests, although there was a trend for less distance travelled by cytokines that was absent in mice pre-treated with the GPR120 agonist. This could be due to an insufficient dose and the high degree of variability in data across mice; nevertheless, locomotion was diminished by cytokines in the social interaction test and prevented in mice pre-treated with CpdA. Cytokine administration also reduced entries into the stimulus mouse zone of the 3CT test, which was absent in the CpdA condition. The duration of time spent near the stimulus mouse unexpectedly increased with cytokine administration, but as this was reversed by CpdA we speculate that the sickness-related actions of the cytokines were at play here. Collectively, these behavioral results are mostly in line with our earlier findings showing central infusions of a GPR120 agonist abrogates anxiety-like behavior in diet-induced obese mice [12]. The longer duration of ICV agonist infusions and inflammatory nature of the diet manipulation may explain the more robust anxiolytic effects of GPR120 stimulation observed in that study. That the behavioral impact of central cytokines was weaker than that obtained with LPS in the current study may well be due to dosing and/or the more robust influence peripheral immune activation can have on the CNS via enhanced production of local inflammatory mediators, not limited to IL-1β and TNF-α, by endothelial cells, perivascular macrophages, microglia and astrocytes at the blood–brain barrier [43–46].

Comorbidity of metabolic and psychiatric diseases have been tied to the immunometabolic consequences of poor diet and obesity development that includes gut dysbiosis, adipose-derived inflammation, metabolic dysfunction and neuroinflammatory consequences [2]. GPR120 is a promising anti-obesity and anti-diabetes target as it mediates FFA signaling in the periphery to regulate insulin and glucagon-like peptide 1 [9]. Along with our previous findings showing that prolonged central GPR120 activation can inhibit anxiety in high-fat fed mice [12], our current results suggest that stimulating brain GPR120 with an agonist that crosses the blood brain barrier may offer a promising strategy for attenuating neuroimmune responses, sickness-like behaviors and mood perturbations via its capacity to restrain microglial reactivity and cytokine synthesis and release. In agreement, peripheral GPR120 activation was also shown to reduce prostaglandin D2-microglia-provoked neuroinflammation and contribute to memory function [47]. n-3 PUFA deficiency heightens anxiety-like behavior, anhedonia and long-term depression of evoked excitatory postsynaptic potential in NAc [48]. In clinical studies, lower n-3 PUFA links with emotion and cognition deficits while n-3 PUFA supplementation offers the potential to improve these changes [49, 50]. While more research is needed to uncover how central GPR120 contributes to the neurobehavioral effects of modulating dietary n-3 PUFA, GPR120-based pharmacotherapies may offer a new management strategy for metabolic disease and psychiatric and neurological co-morbidities that encompass microglial activations. Further studies will be required to elucidate the role of GPR120 activation in the resolution of obesity and associated metabolic inflammation.

## Data Availability

The data that support the findings of this study are available from the corresponding author upon reasonable request.
